# Hydrogels Based Drug Delivery Synthesis, Characterization and Administration

**DOI:** 10.3390/pharmaceutics11090432

**Published:** 2019-08-23

**Authors:** Anca Onaciu, Raluca Andrada Munteanu, Alin Iulian Moldovan, Cristian Silviu Moldovan, Ioana Berindan-Neagoe

**Affiliations:** 1 Medfuture—Research Center for Advanced Medicine, “Iuliu Hațieganu” University of Medicine and Pharmacy, Marinescu 23/Pasteur 4-6 Street, 400337 Cluj-Napoca, Romania; 2 Department of Pharmaceutical Physics-Biophysics, Faculty of Pharmacy, “Iuliu Hațieganu” University of Medicine and Pharmacy, Pasteur 6 Street, 400349 Cluj-Napoca, Romania; 3 Research Center for Functional Genomics, Biomedicine and Translational Medicine, “Iuliu Hațieganu” University of Medicine and Pharmacy, Marinescu 23 Street, 400337 Cluj-Napoca, Romania; 4 The Oncology Institute “Prof Dr Ion Chiricuța”, Republicii 34-36 Street, 400015 Cluj-Napoca, Romania

**Keywords:** hydrogels, drug delivery, target therapy, polymers, biocompatibility, in vivo administration

## Abstract

Hydrogels represent 3D polymeric networks specially designed for various medical applications. Due to their porous structure, they are able to swollen and to entrap large amounts of therapeutic agents and other molecules. In addition, their biocompatibility and biodegradability properties, together with a controlled release profile, make hydrogels a potential drug delivery system. In vivo studies have demonstrated their effectiveness as curing platforms for various diseases and affections. In addition, the results of the clinical trials are very encouraging and promising for the use of hydrogels as future target therapy strategies.

## 1. Introduction

Hydrogels are three-dimensional (3D) polymeric networks, whose hydrophilic structure allows the absorption of large amounts of water (thousands of times their dry weight) [1]. Hydrogels consisting of synthetic polymers are currently gaining more interest than natural-derived polymers due to their enhanced life-time, higher capacity to absorb water [2], improved mechanical properties [3] and finely-tuned degradation [4]. Depending on their structure, hydrogels can be chemically stable or easily degradable [5]. According to the type of cross-linking between the polymers, hydrogels can be classified in physical, held together by non-covalent, reversible interactions and chemical hydrogels, linked by non-reversible covalent bonds [4].

**Figure 1:**
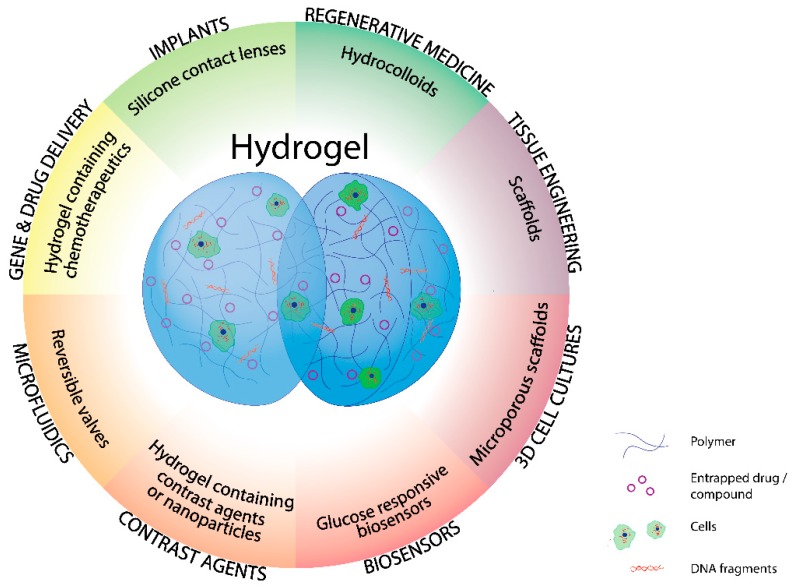
Hydrogel medical applications.

Their unique properties, including reliable biocompatibility, tunable mechanical and degradation features, sensitivity to various stimuli and the ability to be easily conjugated with hydrophilic and hydrophobic therapeutic compounds [4] has made them important candidates in biomedical applications including drug delivery [6], tissue engineering [7,8,9], 3D cell cultures [10], in vitro diagnostics and stem cell research [1] (Figure 1).

In the therapeutic area, drug delivery approaches require outstanding improvements in obtaining safe transport systems in order to achieve the desired therapeutic effect and to avoid the side effects. Biomimetic strategies involving polymers describe innovative industrial products orientated to target therapy and controlled release. Hydrogels inspired design offer favorable conditions for therapeutic compounds encapsulation and protection. Furthermore, they are becoming biological responsive structures which ensure adequate biocompatibility and biodegradability.

## 2. Hydrogel Synthesis Methods

Hydrogel synthesis is an essential step in developing new structures with beneficial properties for drug delivery action. The hydrogel structure is defined by the hydration of hydrophilic groups and domains of the polymers involved. Therefore, these groups and their interconnected chains create three-dimensional networks via crosslinking, avoiding their dissolution in the aqueous phase [2,11]. 

The standard synthesis procedures imply polymerization and crosslinking. These techniques can happen in parallel in one step, or one after the other in multiple steps [2]. 

Polymerization process is part of the gelation process. The structure and the conformation of the starting material influence the formation of soluble branched polymer networks [12]. The starting material refers to polymer monomers, prepolymers, or hydrophilic polymers [13]. The monomers and polyfunctional comonomers act as crosslinkers in network development. Sometimes polymerization is generated by radical initiators [14,15] or photoinitiators [16,17] like irradiation [18,19,20]. 

Hydrophilic polymers are often used for hydrogel synthesis due to their biocompatibility [21,22] in aqueous environments [23] and mostly due to their loading drug potential [24]. 

The swelling property of a hydrogel is significant for further use in medical applications because most of the body tissues are composed of large amounts of water [25]. Swelling features of various polymers are advantageous for functionalization with therapeutic agents. On the other side, the effectiveness of such systems could consist of their capacity to deliver these agents without side outcomes. 

**Figure 2:**
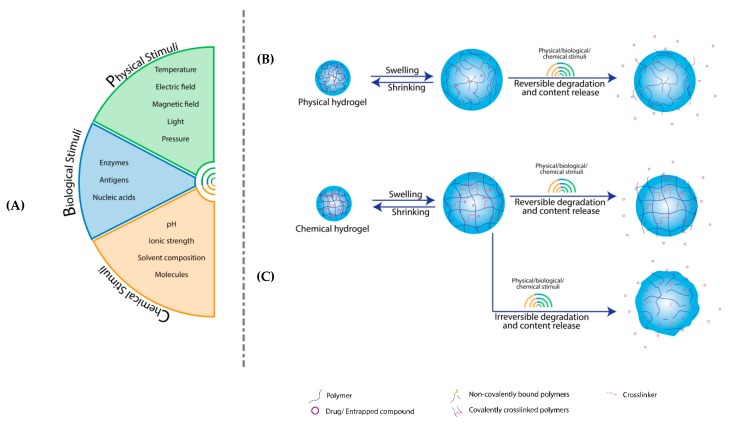
Stimuli sensitive hydrogels structural changes. (**A**) Stimuli categories: physical, biological and chemical. (**B**) Physical hydrogels are non-covalently crosslinked and in an aqueous environment, they swell. Under various stimuli presence, they undergo reversible structure modifications and release the compound. (**C**) Chemical hydrogels describes covalently crosslinked formulation that swells in aqueous conditions and then suffers reversible or irreversible alterations depending on stimuli presence and crosslinking strength, which influence their discharge.

The crosslinker agent plays a significant role in hydrogel swelling [26] and degradation [27]. It influences the physical properties of the final hydrogel product [28]. Crosslinking methods imply covalent or non-covalent interactions between polymer monomers providing elastic characteristics [2,11,29,30]. For this reason, two different types of hydrogels were identified: chemical gels based on covalent interactions (Figure 2C) and physical gels based on non-covalent interactions [11] (Figure 2B).

On the other hand, there are factors involved in altering hydrogels assembly [31]. Chemical stimuli (pH [32,33], ionic strength [34,35], solvent composition [36], and molecules [37]) (Figure 2A) lead to permanent gels. Physical stimuli (temperature [38], electric field [39], magnetic field [40], light [16,41], pressure [42]) (Figure 2A) determine the reversible conversion between un-swollen and swollen state called phase transition. Biological stimuli (enzymes [43], antigens [44], and nucleic acids [45]) (Figure 2A) affect the physical properties of the hydrogels like solubility [46]. These stimuli-responsive polymers belong to the group of intelligent or smart materials [47,48].

Smart polymers can form self-assembly structures through two significant procedures: using peptide/protein materials or using hybrid systems containing minimum a synthetic macromolecule and a peptide/protein motif [49]. Their synthesis includes supramolecular chemistry and the control of the dynamic of supramolecular interactions and bonds for future media conditions [50]. Proteins and peptides extend the system properties to degradability, stimuli-responsive phase transition, and also biological molecules targeting [51]. Such smart hydrogels own convenient characteristics for drug delivery systems through their structural, morphological, and functional modifications occurred due to stimuli detection [52]. These modifications ensure a time-dependent controlled release of the therapeutic agent with long circulation time converging to minimal side effects [53].

## 3. Classification 

Hydrogels can be classified considering different parameters like: polymer origin, ionic charge and biodegradability [54].

According to the polymer origin, the hydrogel composition can be synthetic, natural or it can contain both synthetic and natural polymers and is called hybrid [55]. Lately, the synthetic hydrogels are preferred due to their high water absorption properties, long life, and also a variety of chemistry which confers better strengthens and resistance in different conditions [2]. 

Ionic charge influences the uptake of the charged drug molecules. A positively charged hydrogel will embed and release negatively charged low molecular weight molecules [56]. Furthermore, the ionic charged hydrogels have implications on cellular interactions and behavior [57].

Biodegradable hydrogels need to meet some specific features as biocompatibility and adaptability to various media conditions and preparation procedures. Such hydrogels are potential candidates for drug delivery applications because of their flexibility. Biodegradation process describes a chemical decomposition of a complex hydrogel into a simple structure through solubilizing or hydrolysis dictated by various external stimuli like pH, ionic strength, temperature or enzymes [27]. Therefore, these hydrogels can be divided into chemical, physical and biological stimuli responsive categories.

Otherwise, non-biodegradable hydrogels offer multiple possibilities for tissue engineering applications by encapsulating growth factors, proteins [58]. Other medical fields that are exploiting these type of hydrogels are plastic and reconstruction surgery, orthopedic implants, ocular lens, and microfluidic devices [59]. 

Table 1 summarizes important classes of hydrogels and their properties and applications.

## 4. Hydrogel Functionalization with Therapeutic Agents

Crosslinking synthesis methods make possible the functionalization with drugs and other therapeutic agents in order to develop new delivery systems [121]. The hydrogel design describes cross-linked polymers and meshes that allow compound solution loading and diffusion. When functionalizing, the size of the meshes is to be taken into consideration [122]. This procedure can be performed at two different times of hydrogel synthesis: at the beginning by mixing the drug with the other reagents or at the end after hydrogel is done [29]. In situ loading method suits for hydrophilic drugs and is based on dissolving the drug into the water together with the polymer powder. The other technique is called post-loading and refers to dry hydrogel films immersion into drug solution for a certain period of time. In both of these cases, after drug incorporation, the hydrogel has a dried state and confers protection. In addition, cross-linkers are essential factors in controlled release of high or low molecular weight therapeutic agents, and in most cases the degradable cross-linkers are preferred [56]. 

Wong and Dodou, 2017, synthesized poly(ethylene oxide) hydrogels cross-linked with pentaerythritol tetra-acrylate using ultraviolet light for cross-linking reaction. These hydrogel films were loaded with various drugs (lidocaine hydrochloride, diclofenac sodium and ibuprofen) using post-loading and in situ loading methods. The study results revealed that in situ loading procedure was more successful regarding drug encapsulation [123].

Prince et al., 2019, used in situ loading technique for thermoresponsive poly(ϵ-caprolactone-co-lactide)-block-poly(ethylene glycol)-block-poly(ϵ-caprolactone-*co*-lactide) hydrogels functionalization with celecoxib. PEG’s length affects hydrogel loading capacity and 2000 g/mol was found to be the optimal length for drug delivery applications [124].

Many drugs and small molecules, especially the hydrophobic ones, can be encapsulated in order to enter the gel for further medical applications. For instance, many nanosystems (metallic [125], lipid [126], polymeric [127], peptides [128]) are used for targeted delivery [129]. Nanoparticles are fashioned to ensure drug pharmacokinetics and pharmacodynamics [130].

The use of nanoparticles can help the crosslinking reaction by adsorbing or attaching to polymer chains [131], but also they can modify the hydrogel assembly properties [132]. Due to the porous hydrogel structure, nanoparticles are easily embedded within a polymeric 3D network. This aspect is mandatory for achieving controlled release profile and can impede in choosing the right nanoparticles for drug molecules delivery, respectively those for hydrogel functionalization [133]. Other nanoparticle physico-chemical properties like size, polydispersity index, and spatial orientation inside the gel need to be carefully adjusted [134]. 

High loading capacity combined with controlled drug release and prolonged stability, will represent advantages for the newly designed system [135]. 

Nanoparticle functionalized gels can perform targeted drug delivery in different ways: passive [136], stimuli-responsive [137], site-specific [138] or detoxification manner [139]. 

## 5. Characterization Methods

When considering hydrogel characteristics usually investigated, the typical protocol will most probably compose of two kinds of tests: structural and functional. For structural analysis, an assortment of microscopy techniques have become, to a degree, the golden standard. As hydrogels are three dimensional, commonly optically clear, materials, conventional bright field microscopy is sparsely used. By far, the most common technique is Scanning Electron Microscopy (SEM). An extensive review of morphological characterization of hydrogels has been recently published [140], and it implies that an SEM is a go-to instrument for hydrogel characterization regardless of composition. SEM imaging does require specialized sample preparation instrumentation, but the methodology was standardized to most gel types. Briefly, formalin fixed samples are dehydrated and freeze-dried, followed by either Au or Au/Pd sputtering under vacuum before imaging [141]. Newer protocols forgo the fixation and dehydration steps [142] even though there is evidence that liquid nitrogen snap freezing followed by freeze-drying induces gel shrinkage and incorrect evaluation of pore size [143,144]. SEM is generally used to asses pore formation and pore size [145], crosslinking status [146] and the effect certain loading compounds have on general gel structure [147].

While SEM does provide valuable qualitative information, alternatives do exist and can potentially provide complementary information to the limited capacity of regular SEM of generating two-dimensional projections. Laser scanning confocal microscopy (LSCM), used in combination with fluorescent dyes is capable of generating Z-stacks to evaluate similar aspects (i.e., pore dimensions and shape) to SEM [140,148]. Where LSCM shines is the complementary characterization capacity it has. Hydrogel loading and unloading of solutes [149], dispersion and mobility of the solute [150] or distribution of solid loading materials [151] are tested with the condition that the solute (or other compounds of interest) is fluorescent or can be spiked/bound to a fluorescent marker. Structural characteristics, along with mechanical proprieties, can also be observed by different variations of Scanning Probe Microscopy. It was proved that the elastic modulus of the gel correlates is correlated with cell differentiation [152] and migration [153]. As the modulus of the hydrogel correlates with the degree of polymer cross-linkage [154] and cross-linkage to solute mobility through the gel [155], Atomic Force Microscopy is a powerful multimodal analysis tool in the characterization of hydrogels, allowing not only topological and roughness investigations but also functional ones. Of course, all the techniques presented can be used in parallel or correlatively to offer complementary information or potentially highly specific characteristics such as individual pore mechanics [156]. 

Functional analysis is paramount to understanding the effects that solute/external stimuli have on the hydrogel. The most important ones seem to be: absorption capacity and rate, absorbency under load, lowest solute, and extra monomer level, pH, and photostability [2]. Drug release from the hydrogel is usually a function of the diffusion capacity of the drug and the retention capacity of the gel. This is most commonly measured by dynamically probing the concentration of the drug in the medium of interest and plotting the concentration against a certain time interval or as a percentage. The actual analysis is highly dependent of the nature of the tested compound and can range from HPLC for peptides [157], electrochemical probing for gases such as H_2_S [158] or various fluorescence/colorimetric/absorbancy tests. 

The controlled release can be achieved through various strategies based on hydrogel composition and properties. For instance, there are some molecules that can initiate and enhance the degradation rate [159,160]. Matrix-metalloproteinases family members can cleave the oligo-peptide bonds [161]. Other examples are represented by glucose [162] and thrombin [163], which are playing important roles in physiological processes. On the other hand, external stimuli [164,165] can determine hydrogel networks disruption. Hydrogels swelling capacity is an important target for the sustained release mechanism [166] and is also influenced by stimuli sensitivity [167]. 

In addition, hydrogel mechanical deformation using magnetic field or ultrasounds is a very common procedure. In this regard, Liu et al., 2006, proposed an intelligent magnetic hydrogel loaded with drug molecules capable of tunable controlled release profile time dependent [168]. Ultrasound guided drug release approach was digitally integrated into ionically crosslinked hydrogels [169].

Many mathematical models are used for predicting the drug delivery efficiency of polymeric hydrogels. These determinations are based on mesh size, mechanical, mass-transport and diffusion behavior using hydrodynamics, obstruction, free volume and combined theories [166,170].

## 6. In Vivo Biocompatibility and Biodegradability

In order to compare and investigate the effects of hydrogels in vivo, it is necessary to evaluate its biocompatibility, since living organisms are prone to develop inflammatory reactions which are facilitated by the degradation of the synthetic polymers [171]. As stated in a review by Naahidi et al., hydrogel toxicity and biocompatibility is dependent on the breakdown of the polymer into monomers or oligomers, the crosslinking agents or trace polymerization agents [172]. Besides achieving favorable hydrogel formulations for drug delivery, the main challenges are performing toxicity screening [173], maintaining long-term stability, and controlling the release properties of the therapeutic agents. One favorable aspect of hydrogel research has been its longevity. The over 100 years of research have produced gel-like biopolymers with low toxicity and high biocompatibility, especially those derived from natural molecules such as collagen, chitosan, fibrin, and hyaluronic acid [174]. However, while previous in vitro studies have shown promising results regarding the biodegradability and biocompatibility of these compounds, further in vivo studies are necessary at this point [175]. 

## 7. Hydrogel Administration

**Figure 3:**
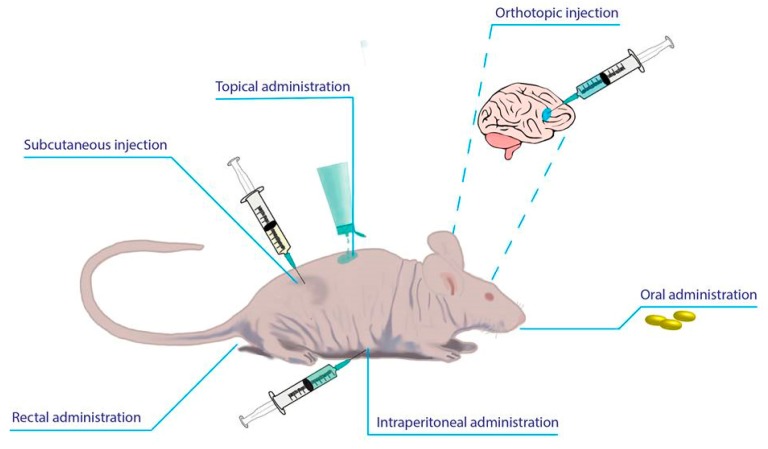
In vivo drug delivery hydrogel routes of administration. There are various methods for in vivo administration of hydrogel based products depending on the pathological conditions and their localization. Subcutaneous injection plays a crucial role in toxicological effects evaluation. Topical or transdermal application is preferred for skin associated problems. Orthotopic and intraperitoneal injections are non-invasive techniques which ensure good therapy results. On the other hand, oral administration has some limitations because of digestive enzymes.

As mentioned above, a complex problem regarding the administration of various biomaterials in vivo reflects the ability of the material to conserve and promote a biologically safe environment for the subjected animals. In vivo testing is challenging because there still is a need for an established and reliable animal model in order to achieve biomechanical restoration. Following these standards, different injectable hydrogels protocols were developed and optimized, which because the body very well tolerated them, are ideal candidates for performing in vivo testing on rodents [176]. Given the fact that these delivery systems are suitable for clinical use, the most accessible routes of administration are subcutaneous [177], oral [178], rectal [179], topical and transdermal [180], orthotopic [181], intraperitoneal [182] and ocular [183]. Figure 3 represents all these administration routes.

### 7.1. Subcutaneous Hydrogel Delivery System

One of the most efficient methods to evaluate the response to the therapy and assessing the toxic response in vivo in mice is performing a subcutaneous injection. Since the subcutaneous area is vascularized, the implanted hydrogels or other biomaterials are immune privileged, so a mild reaction to foreign moieties is to be expected [176]. Bare polyethylene hydrogels have been proven to not exhibit cytotoxicity in murine models even after 60 days when injected subcutaneously [184]. Similar results regarding biocompatibility were obtained with ellagic acid hydrogels [185], nano-patterned poly-acrylamide hydrogels [186] chitosan and gelatin hydrogels [187,188], alginate [189] and pectin [190]. It is worth mentioning that the majority of the studies do report mild inflammatory responses.

### 7.2. Oral Delivery

The oral delivery route represents by far the most convenient solution under adjustable parameters and patient compliance [191]. Orally administered hydrogels should provide bioavailability depending on medium particularities, such as pH variations along the intestinal tract [192]. MPEG, caprolactone and, itaconic acid pH-sensitive hydrogels were tested for acute oral toxicity in BALB/c mice and showed no signs of toxicity [193]. When considering oral ingestion, the metabolic effect that monomers have on the organism is very significant. Poly-glycolic and poly-lactic-*co*-glycolic acid hydrogel degradation have been shown to affect healthy metabolism [172], leading to some limitations in terms of hydrogel therapy such as:

1. Presence of digestive enzymes could lead to denaturation;
2. Low permeability through the epithelial membrane into the bloodstream;
3. Superior and inferior digestive systems can represent potential therapeutic targets [194].

### 7.3. Rectal Delivery

Rectal administration offers plenty of advantages such as rapid absorption of the compound, avoidance of the gastrointestinal tract, limited adverse reactions of the therapeutics, and provides controlled release of the compound in the context of the stable environmental conditions encountered in the rectum. Based on an earlier study that showed excellent biocompatibility on the digestive tract [195], rectally administrated catechol-chitosan- based hydrogels with mucoadhesive properties have been tested in murine models and show no toxic effects after 10 days [196]. Regarding this issue, Afaf A. et al., developed a hydrogel-based product and made a correlation between in vitro and in vivo profile with promising results [179].

### 7.4. Topical and Transdermal Delivery

Hydrogel therapy is suitable for the topical and transdermal approach. Nowadays, the general concept about nanosystems in the context of skin penetration is that the skin barrier restricts the delivery via epidermis and dermis. In truth, hydrogel penetration through the skin seems unlikely as the macroscale mesh-like structure would prohibit penetration and has been shown, in the case of collagen hydrogels, to not even affect burn models [197]. Various alternative hydrogel formulations are commonly tested on graft donors with no discernable side effects compared to other gel-based formulations commonly used in hospital environments [198]. Recent studies demonstrated that this method of delivery is reliable as a delivery system for nanoparticles with various roles, and it only depends on nanoparticles diameter, charge, and structure. The coating influences the penetration rate. Peptide layered nanocarriers are more efficient than pegylated ones. Ex vivo studies were carried out, using skin samples collected from mouse and human through surgical resection [199]. 

### 7.5. Orthotopic Injections

Administration of hydrogels via intratumoral injection is a practical approach, which requires the release of the nanostructure system loaded in a hydrogel matrix. These hydrogel structures are so-called “macroscopic gels” that are coming with the imperative need to decrease potential damages of associated tissues during an injection [200]. Qinjie Wu et al., 2015 synthesized a smart hydrogel responsive to temperature, which prevented the formation of peritoneal adhesion on a damaged abdominal wall [181]. Where orthotopic treatment using hydrogels seems to show promise is in the post-resection cavity where it can act as a slow release to improve long term survival [201,202].

### 7.6. Intraperitoneal Delivery

Intraperitoneal administration of hydrogel systems shows promising results and is considered to be a noninvasive option, as well as an optimal formulation for various pharmaceutical agents. One of these structures was prepared by Chih-Hao Chen et al., in 2018 and was successfully incorporated in the mouse peritoneum achieving dual action: drug delivery and postoperative anti-adhesiveness [203]. Similarly to orthotopic injected hydrogels, intraperitoneal hydrogels (mostly gelatin based gels) by themselves show no cytotoxicity and high degrees of biocompatibility [204]. It is worth mentioning that the capacity of the gels to absorb high amounts of water could be detrimental in the case of any injectable formulations [182]. 

### 7.7. Ocular Delivery

Formulations for ocular delivery are complicated to develop; several implications are creating limitations like inferior bioavailability and absorption [183]. Eye topical applications can be performed but with minimal disease addressability. Delivering therapeutic agents in maximum concentrations needed in the posterior segment encounters failure in most of the cases [205]. However, for a better approach of the posterior segment, intraocular injections are more operative, but in this case, ocular complications are the primary concern. There are still other approaches like intravitreal, intracameral or peri-ocular injections, but with a significant risk of side effects [206]. Improvements in protocols alongside adaptations of in vivo performance for sustainable results are needed. The standard hydrogels used for contact lenses (silicone and polymethylemethacrylate) are, however, generally highly biocompatible with almost 50 years of results proving it [207]. Some issues with silicone hydrogels causing eye inflammation have been reported [208] but, the only specific limitation to capable hydrogel based therapeutics seem to be those mentioned above. 

### 7.8. Tissue Engineering and Bone Repair

Innovative biocompatible and biodegradable injectable hydrogels have been synthesized for cartilage and bone tissue [209], vascular tissue, skin, and other soft tissues engineering [210]. In this regard, the viscosity of the hydrogel plays a significant role together with sol-gel transition in specific physiological environment [210]. 

The injectable systems have the flexibility to fill irregular-shaped defects and offer the possibility to reduce surgical invasion [211]. Significant current research strategies have been reported regarding the use of autografts in bone implantation therapy, due to the advantage of minimized rejection rates [212]. Recent attempts have been made in injectable bio hydrogels (collagen [213], chitosan [214], gelatin [215], cellulose [97]) in cell-based therapies to increase biomimetic capacity. For instance, Park and Lee developed an oxidized alginate/hyaluronic acid hydrogel construct and tested it together with chondrocytes in vivo on mice, and obtained positive results on cartilage regeneration [216]. 

Among these, stem cell delivery systems are focusing on microfluidics to avoid microenvironment interactions. Various hydrogel constructs like microspheres were fromulated for stem cells encapsulation. For example, Zhao et al., 2016, synthesized light responsive hydrogel microspheres made by gelatin and methacryloyl chloride. Bone marrow-derived mesenchymal stem cells were encapsulated using post-loading procedures and then tested in vivo on rabbit femoral condyle animal model following orthotopic implantation protocol. This microfluidic approach demonstrates the feasibility of this method in osteogenesis [217]. 

In situ gel forming tissue implants are suitable for transplantation during invasive surgeries directly into defects. The difficult regeneration of articular cartilage represents a very challenging problem. Due to the fact that this tissue is not presenting vascularization, the immune response is absent and it allows the use of allogenic constructs in the context of tissue engineering matter. Perrier-Groult et al., 2019, implemented a protocol for subcutaneous administration of agarose hydrogels embedded with human chondrocytes in order to promote their differentiation to cartilage matrix. In this regard, humanized mouse models are preferred for the evaluation of such implants acceptance [218]. 

Considering the aspects mentioned above, when choosing the experimental design for in vivo studies using murine models, the age of the animal should be considered because it can influence the outcomes through various metabolic pathways involved in hydrogels degradation and clearance [175]. 

Regarding hazard assessment, many different hydrogel systems aim to deliver the therapeutic agent in a controlled manner for better effects at the target site [219]. In a general approach to a pathology, orthotopic murine models simulate clinical conditions with better accuracy than cell line-based models, due to proper exposure to a microenvironment that is more similar to the pathological status in human [220]. Therefore, orthotropic animal models should be implemented in order to improve the response to therapy significantly.

## 8. Clinical Trials 

Bringing together the advantages of a biocompatible hydrogel with the therapeutic effect of various molecules provides new efficient configurations adapted for medical devices development. Polymer science is holding up the attention of scientists because of the promising properties of biomedical clinical applications. 

In the last decade, an increasing number of clinical trials investigated the use of hydrogel structures for drug delivery systems. According to ClinicalTrials.gov, over 300 completed studies are presenting encouraging results for hydrogel use with significant applicability in visual disorders and dermatological affections. Table 2 emphasizes prominent examples of such products.

## 9. Future Perspectives

In the frame of current work, hydrogels aim to become feasible tools for microfluidics, biosensing and tissue regeneration engineering (potential scaffold for new tissues and organs). Future proposed designs highlight the control over structural and morphological properties in order to improve their biomimetic capabilities. 

Furthermore, hydrogel usage in generating complex 3D multi-cell models offers promise in reducing the number of animals used for testing and while at the same time provides medium that more closely resembles aspects of the human body. All of these considerations will inevitably lead to accelerating pharmaceutical formulation development and deployment on the market. It is truly difficult to exemplify the role and applications that hydrogels have in biomedical research mainly due to their usage not being restricted to the topics described in this review. It is, however, easy to observe the capacity that this technology has to transcend a certain niche and in a very timely manner be implemented in a completely new field of work. The most difficult hurdles for medical and biological research seem to have been overcome and hydrogels have become a common day item in not only researchers’ lives but the general public’s lives too. 

Recently, electronics progress relieves the use of hydrogel technology for healthcare oriented applications. Lab-on-a-chip microfluidic devices are considered practical and compelling for diagnostic and drug screening investigations. The generation of wearable sensors represents an innovative platform able to monitor pathological particularities of the human subject. Such devices can also perform a controlled release of pharmaceutical formulations according to the monitored parameters.

What is clear is the fact that hydrogels have been and are continuing to be relevant “scaffolds” in biomedicine and biomedical research. When it comes to these constructs we have entered an era of fine-tuning already existing structures and technology, where novel hydrogels are quick to reach practical applications in the real world (contact lenses, wound dressing, 3D culture scaffolds) and more exotic ones hold potential in a variety of fields such as robotics, aerospace engineering, solar cells and photoreactors, environmental research and sports science.

## Figures and Tables

**Table 1:** Hydrogel classification according various parameters, their properties and corresponding applications.

Parameter	Hydrogel Type	Hydrogel Composition	Properties	Applications
Chemical stimuli	pH responsive	Carboxylated agarose/tannic acid hydrogel scaffolds cross-linked with zinc ions [60] Poly(acrylamide-*co*-acrylic acid) poly(AAm-*co*-AAc) hydrogels [61]	Sustained release of the incorporated drugs [60] Biocompatibility [60] Strong electrostatic interactions and stability [61] Increased hydrophilicity and swelling [62]	Drug delivery [54,60] Sensing [63]
	Ionic strength responsive	2-acrylamido-2-methylpropane sulfonic acid crosslinked with *N*,*N*’-methylene(bis)acrylamide [34] Poly(*N*-isopropylacrylamide) crosslinked with imidazolium-based dicationic ionic liquid [64]	Increased swelling properties [34] Controllable porous structure [34] Biodegradability [65]	Depollution of aqueous ecosystems [64] Drug and gene delivery [47] Tissue engineering [47]
	Solvent composition responsive	Fluorenylmethoxycarbonyl diphenylalanine [36] Poly(*N*-isopropylacrylamide) and poly(*N*,Ndimethylacrylamide) mixtures [66] Poly(*N*-isopropylacrylamide) [67,68]	Uniform networks [36] Swelling behaviour responsive to temperature too [66] High porosity, Heterogeneous structure [67]	Sensing [68]
	Molecules responsive	*N*-isopropylacrylamide crosslinked with *N*,*N*′-methylenebis(acrylamide) [69] Acrylamide crosslinked with polyethylene glycol [70]	Achieves molecular recognition, High affinity and specificity [71] Controlled assembly [72] Controlled release [43] Enzyme responsive [70] Antigen responsive [69]	Sensing [73] Drug delivery [37] Cell culture [72]
Physical stimuli	Temperature responsive	*N*-trimethyl chitosan chloride polymers crosslinked with poly(ethylene glycol) and glycerophosphate [74] Poly(*N*-vinylcaprolactam) grafted with poly(ethylene oxide) [75] Poly(*N*-isopropylacrylamide) and aminated alginate [76] Poly(*N*-vinylcaprolactam) [77] Methoxy poly(ethylene glycol)-poly(pyrrolidone-*co*-lactide) [78]	Two categories: low critical solution temperature [74] and upper critical solution temperature [75] Sol –gel transition at 37 °C [79] Easy functionalization with drug molecules [80] Unique physical properties similar to extracellular matrix [81] Controlled degradation [76]	Tissue engineering [76,77,78,82], Drug delivery [80,82]
	Electric field responsive	Polypyrrole polymeric nanoparticles loaded in poly lactic-*co*-glycolic acid and poly(ethyleneglycol) hydrogel [83]	Controlled release of the cargo [84] depending on the strength or the duration of applied electric field [83] Biocompatibility, Minimal invasiveness [83]	Drug delivery [84]
	Magnetic field responsive	Hemicellulose crosslinked with O-acetyl-galactoglucomannan [85] Gelatin hydrogels loaded with magnetic nanoparticles [86]	Successful absorption and controlled release of drugs [85] Some of them dispose of anisotropic properties [87]	Tissue engineering [86] Microfluidics, drug delivery, contrast agents [88]
	Light responsive	Hydroxypropyl methylcellulose and Carbopol hydrogels containing diclofenac-sodium chitosan microspheres [89] Poly[2-((4,5-dimethoxy-2-nitrobenzyl)oxy)-*N*-(2-(methacryloyloxy)ethyl)-*N*, *N*-dimethyl-2-oxoethan-1-aminium] [90]	Reasonable strengthens according to application [89] Reversible and irreversible, Spatiotemporal control over functional groups, Controlled release [91]	Drug delivery [89] Self-sterilization and self-cleaning [90] Microfluidics [92]
	Pressure responsive	Polyacrylamide and poly(acrylamide-hydroxyethyl methacrylate) [93]	Thermo- and pH sensitive [94] Adhesion capacity, elasticity [93]	Sensing [95] Drug delivery [96]
Polymer origin	Natural	Nanofibrillar cellulose [97] Thiolated gelatin-poly(ethylene glycol) diacrylate [98] Methacrylated gelatin [99] Polycaprolactone sandwiched in a gelatin-chitosan hydrogel [100]	Biomimetic and adhesion capacity [101] Mechanical support for cell development [102]	Tissue engineering [101,103,104], Drug delivery, Sensing [102]
	Synthetic	Low acyl gellan gum bilayered hydrogel scaffolds [105] *N*-isopropylacrylamide and itaconic acid [106] Poly(ethylene glycol)—poly(propylene glycol) copolymers [107]	Controllable structure and other physico-chemical properties [102] Stimuli responsive [106]	Drug delivery [106] Tissue engineering [108]
	Hybrid	Alginate-polymethacrylate [109] Chondroitin sulfate and poly(ethylene glycol) [110] Chitosan/hyaluronic acid hydrogels loaded with poly (lactic-*co*-glycolic acid) microspheres [111]	Biomimetic capacity [109] Multicomponent [112] Heterogeneous composition [113] Responsive to environment changes [114]	Tissue engineering, drug delivery [112] Wound-healing [111]
Biodegradability	Biodegradable	Chitosan-gelatin [115] Pectin-*co*-poly(methacrylic acid) [116]	Stable and biocompatible [116] Biomimetic capacity [117] Natural and synthetic polymeric structure [117] Stimuli responsive [118]	Drug delivery [116] Tissue engineering [117]
	Non-biodegradable	Poly(2-hydroxyethyl methacrylate) [58] Poly(2-hydroxyethyl methacrylate)/trimethylolpropane trimethacrylate [119]	Biocompatibility [58] Sustained release and recharge [119]	Tissue engineering [58] Drug delivery [119] Plastic and reconstruction surgery [120]

**Table 2:** Different completed clinical trials using hydrogel based products (ClinicalTrials.gov).

Condition	Product	Benefits	Reference
Prostate cancer radiotherapy	Hydrogel spacer	Minimal side effects and toxicity Improves rectal dosimetry Reduces the rates of rectal toxicity	[221,222]
Gynecologic laparoscopic surgery	Crosslinked hyaluronan gel	Safety use Minimizes postoperative adhesion formation throughout the abdominopelvic cavity	[223]
Corneal epithelial permeability	Silicone hydrogel contact lenses	Improves epithelial permeability when used with ophthalmic solutions	[224]
Corneal infiltrates		Identification of bacterial species during continuous wear of contact lenses Improved cornea response to contact lenses	[225,226]
Myopia		Good ocular comfort High oxygen transmissibility	[227]
Dry eye syndrome	Crosslinked hyaluronic acid with liposomes and crocin	Safety profile Promotes re-epithelialization	[228]
Urinary incontinence	Polyacrylamide hydrogel	Facilitates urinary incontinence symptoms for patients that are ineligible for midurethral sling surgery Low rate of adverse effects	[229]
Cerebrospinal fluid leak	Fibrin sealant	Efficient adjunct to dural sutures repair Safe profile	[230]
Diabetes, foot ulcer	Hydrogel/hydrocolloid	Promotes wound healing Confers protection Stimulates epithelial migration	[231]
Intracranial aneurysm	Hydrogel coils	Efficient endovascular coil embolization Safe profile	[232]
Cerebral aneurysm		Improves aneurysm packing Decreases major recurrence	[233]
Submucosal tumor of gastrointestinal tract	Calcium-alginate gel	No adverse events and no tissue injuries Increases mucosa-elevating capacity	[234]
Oral mucositis	Mucoadhesive hydrogel	Safety profile and tolerability Reduces oral mucositis symptoms	[235]
Myoma	Resorbable hydrogel	Safety and efficacy Reduces post-operative adhesions formation following myomectomy	[236]
Pulmonary emphysema	Fibrin hydrogel	Safe profile and no major adverse effects Promotes the formation of scar tissue Improves lung function	[237,238]
Lung cancer biopsy	Hydrogel plug	Reduces postbiopsy pneumothorax and other complications associated with CT guided coaxial needle biopsy	[239]
Ischemic cardiomyopathy	Gelatin hydrogel	Controlled release of fibroblast growth factor Increases the formation of cardiovascular networks Improves ventricular function	[240]
Heart failure	Alginate hydrogel	Efficiency and safety profile No serious adverse effects Increases exercise capacity	[241,242]
Elective cranial procedures with dural incision	PEG hydrogel	Safe profile Dural closure augmentation Rapid preparation and application	[243]
Neuropathic pain	Lidocaine plaster	Safety and tolerability profile Relevant pain reduction	[244]

## References

[1] Seliktar D. (2012). Designing Cell-Compatible Hydrogels for Biomedical Applications. Science.

[2] Ahmed E.M. (2015). Hydrogel: Preparation, characterization, and applications: A review. J. Adv. Res..

[3] Sun J.Y., Zhao X., Illeperuma W.R.K., Chaudhuri O., Oh K.H., Mooney D.J., Vlassak J.J., Suo Z. (2012). Highly stretchable and tough hydrogels. Nature.

[4] Toh W.S., Loh X.J. (2014). Advances in hydrogel delivery systems for tissue regeneration. Mater. Sci. Eng. C.

[5] Hoffman A.S. (2012). Hydrogels for biomedical applications. Adv. Drug Deliv. Rev..

[6] Bhattarai N., Gunn J., Zhang M. (2010). Chitosan-based hydrogels for controlled, localized drug delivery. Adv. Drug Deliv. Rev..

[7] Vedadghavami A., Minooei F., Mohammadi M.H., Khetani S., Rezaei Kolahchi A., Mashayekhan S., Sanati-Nezhad A. (2017). Manufacturing of hydrogel biomaterials with controlled mechanical properties for tissue engineering applications. Acta Biomater..

[8] Lee K.Y., Mooney D.J. (2001). Hydrogels for Tissue Engineering. Chem. Rev..

[9] Buduru S.D., Gulei D., Zimta A.A., Tigu A.B., Cenariu D., Berindan-Neagoe I. (2019). The Potential of Different Origin Stem Cells in Modulating Oral Bone Regeneration Processes. Cells.

[10] Langhans S.A. (2018). Three-Dimensional in Vitro Cell Culture Models in Drug Discovery and Drug Repositioning. Front. Pharmacol..

[11] Hennink W., van Nostrum C. (2002). Novel crosslinking methods to design hydrogels. Adv. Drug Deliv. Rev..

[12] Gulrez S.K., Al-Assaf S., Phillips G.O. (2011). Hydrogels: Methods of Preparation, Characterisation and Applications. Progress in Molecular and Environmental Bioengineering—From Analysis and Modeling to Technology Applications.

[13] Schacht E.H. (2004). Polymer chemistry and hydrogel systems. J. Phys. Conf. Ser..

[14] Jeong G.T., Lee K.M., Yang H.S., Park S.H., Park J.H., Sunwoo C., Ryu H.W., Kim D., Lee W.T., Kim H.S. (2007). Synthesis of poly (sorbitan methacrylate) hydrogel by free-radical polymerization. Appl. Biochem. Biotechnol..

[15] Qavi S., Pourmahdian S., Eslami H. (2014). Acrylamide Hydrogels Preparation via Free Radical Crosslinking Copolymerization: Kinetic Study and Morphological Investigation. J. Macromol. Sci. Part A.

[16] Sawhney A.S., Pathak C.P., Hubbell J.A. (1993). Bioerodible hydrogels based on photopolymerized poly (ethylene glycol)-co-poly (.alpha.-hydroxy acid) diacrylate macromers. Macromolecules.

[17] Haraguchi K., Takada T., Haraguchi R. (2018). Nanocomposite Gels by Initiator-Free Photopolymerization: Role of Plasma-Treated Clay in the Synthesis and Network Formation. ACS Appl. Nano Mater..

[18] Rosiak J.M., Ulański P. (1999). Synthesis of hydrogels by irradiation of polymers in aqueous solution. Radiat. Phys. Chem..

[19] Ikada Y., Mita T., Horii F., Sakurada I., Hatada M. (1977). Preparation of hydrogels by radiation technique. Radiat. Phys. Chem..

[20] Ghobashy M.M. (2018). Ionizing Radiation-Induced Polymerization. Ionizing Radiation Effects and Applications.

[21] Poon K., Castellino V., Cheng Y.L. (2007). Polymeric hydrophilic polymers in targeted drug delivery. Artificial Cells, Cell Engineering and Therapy.

[22] Schmidt B.V.K.J. (2019). Hydrophilic Polymers. Polymers.

[23] Finch C.A. (1987). Hydrophilic polymers. Specialty Polymers.

[24] Liechty W.B., Kryscio D.R., Slaughter B.V., Peppas N.A. (2010). Polymers for Drug Delivery Systems. Annu. Rev. Chem. Biomol. Eng..

[25] Mitchell H., Hamilton T.S., Steggerda F.R., Bean H.W. (1945). The chemical composition of the adult human body and its bearing on the biochemistry of growth. J. Biol. Chem..

[26] Zhang J., Wang A. (2007). Study on superabsorbent composites. IX: Synthesis, characterization and swelling behaviors of polyacrylamide/clay composites based on various clays. React. Funct. Polym..

[27] Kamath K.R., Park K. (1993). Biodegradable hydrogels in drug delivery. Adv. Drug Deliv. Rev..

[28] Jaya M., Vivek Kumar S. (2014). Cross-linking in Hydrogels—A Review. Am. J. Polym. Sci..

[29] Mishra B., Upadhyay M., Reddy Adena S., Vasant B., Muthu M. (2017). Hydrogels: An Introduction to a Controlled Drug Delivery Device, Synthesis and Application in Drug Delivery and Tissue Engineering. Austin. J. Biomed Eng..

[30] Oyen M.L. (2013). Mechanical characterisation of hydrogel materials. Int. Mater. Rev..

[31] Wei M., Gao Y., Li X., Serpe M.J. (2017). Stimuli-responsive polymers and their applications. Polym. Chem..

[32] Shin J., Braun P.V., Lee W. (2010). Fast response photonic crystal pH sensor based on templated photo-polymerized hydrogel inverse opal. Sens. Actuators B Chem..

[33] Chiu Y.L., Chen S.C., Su C.J., Hsiao C.W., Chen Y.M., Chen H.L., Sung H.W. (2009). pH-triggered injectable hydrogels prepared from aqueous N-palmitoyl chitosan: In vitro characteristics and in vivo biocompatibility. Biomaterials.

[34] Ozmen M.M., Okay O. (2006). Superfast Responsive Ionic Hydrogels: Effect of the Monomer Concentration. J. Macromol. Sci. Part A.

[35] Liu S., Oderinde O., Hussain I., Yao F., Fu G. (2018). Dual ionic cross-linked double network hydrogel with self-healing, conductive, and force sensitive properties. Polymer.

[36] Raeburn J., Mendoza-Cuenca C., Cattoz B.N., Little M.A., Terry A.E., Zamith Cardoso A., Griffiths P.C., Adams D.J. (2015). The effect of solvent choice on the gelation and final hydrogel properties of Fmoc-diphenylalanine. Soft Matter.

[37] Zhao F., Ma M.L., Xu B. (2009). Molecular hydrogels of therapeutic agents. Chem. Soc. Rev..

[38] Liu C.B., Gong C.Y., Huang M.J., Wang J.W., Pan Y.F., Zhang Y.D., Li G.Z., Gou M.L., Wang K., Tu M.J. (2008). Thermoreversible gel–sol behavior of biodegradable PCL-PEG-PCL triblock copolymer in aqueous solutions. J. Biomed. Mater. Res. Part B Appl. Biomater..

[39] Li H. (2009). Kinetics of smart hydrogels responding to electric field: A transient deformation analysis. Int. J. Solids Struct..

[40] Namdeo M., Bajpai S.K., Kakkar S. (2009). Preparation of a Magnetic-Field-Sensitive Hydrogel and Preliminary Study of Its Drug Release Behavior. J. Biomater. Sci. Polym..

[41] Ono K., Saito Y., Yura H., Ishikawa K., Kurita A., Akaike T., Ishihara M. (2000). Photocrosslinkable chitosan as a biological adhesive. J. Biomed. Mater. Res..

[42] Lin G., Chang S., Hao H., Tathireddy P., Orthner M., Magda J., Solzbacher F. (2010). Osmotic Swelling Pressure Response of Smart Hydrogels Suitable for Chronically-Implantable Glucose Sensors. Sens. Actuators B Chem..

[43] Chandrawati R. (2016). Enzyme-responsive polymer hydrogels for therapeutic delivery. Exp. Biol. Med..

[44] Zhang R., Bowyer A., Eisenthal R., Hubble J. (2007). A smart membrane based on an antigen-responsive hydrogel. Biotechnol. Bioeng..

[45] Murakami Y., Maeda M. (2005). DNA-Responsive Hydrogels That Can Shrink or Swell. Biomacromolecules.

[46] Dong Y., Wang W., Veiseh O., Appel E.A., Xue K., Webber M.J., Tang B.C., Yang X.W., Weir G.C., Langer R. (2016). Injectable and Glucose-Responsive Hydrogels Based on Boronic Acid–Glucose Complexation. Langmuir.

[47] Deen G., Loh X. (2018). Stimuli-Responsive Cationic Hydrogels in Drug Delivery Applications. Gels.

[48] Li Z., Shen J., Ma H., Lu X., Shi M., Li N., Ye M. (2012). Preparation and characterization of pH- and temperature-responsive hydrogels with surface-functionalized graphene oxide as the crosslinker. Soft Matter.

[49] Kopeček J., Yang J. (2012). Smart Self-Assembled Hybrid Hydrogel Biomaterials. Angew. Chem. Int..

[50] Ferreira N.N., Ferreira L.M.B., Cardoso V.M.O., Boni F.I., Souza A.L.R., Gremião M.P.D. (2018). Recent advances in smart hydrogels for biomedical applications: From self-assembly to functional approaches. Eur. Polym. J..

[51] Deming T. (2012). Peptide-Based Materials.

[52] Zheng H., Xing L., Cao Y., Che S. (2013). Coordination bonding based pH-responsive drug delivery systems. Coord. Chem. Rev..

[53] Schmaljohann D. (2006). Thermo- and pH-responsive polymers in drug delivery. Adv. Drug Deliv. Rev..

[54] Rizwan M., Yahya R., Hassan A., Yar M., Azzahari A., Selvanathan V., Sonsudin F., Abouloula C. (2017). pH Sensitive Hydrogels in Drug Delivery: Brief History, Properties, Swelling, and Release Mechanism, Material Selection and Applications. Polymers.

[55] Peppas N.A., Hilt J.Z., Khademhosseini A., Langer R. (2006). Hydrogels in biology and medicine: From molecular principles to bionanotechnology. Adv. Mater..

[56] Blöhbaum J., Paulus I., Pöppler A.C., Tessmar J., Groll J. (2019). Influence of charged groups on the cross-linking efficiency and release of guest molecules from thiol–ene cross-linked poly (2-oxazoline) hydrogels. J. Mater. Chem. B.

[57] Hegger P.S., Kupka J., Minsky B.B., Schädel N., Petri N., Laschat S., Boehm H. (2017). Charge Matters: Modulating Secondary Interactions in Hyaluronan Hydrogels. ChemistrySelect.

[58] Takeda K., Kitagawa H., Tsuboi R., Kiba W., Sasaki J.I., Hayashi M., Imazato S. (2015). Effectiveness of non-biodegradable poly (2-hydroxyethyl methacrylate)-based hydrogel particles as a fibroblast growth factor-2 releasing carrier. Dent. Mater..

[59] Shastri V. (2005). Non-Degradable Biocompatible Polymers in Medicine: Past, Present and Future. Curr. Pharm. Biotechnol..

[60] Ninan N., Forget A., Shastri V.P., Voelcker N.H., Blencowe A. (2016). Antibacterial and Anti-Inflammatory pH-Responsive Tannic Acid-Carboxylated Agarose Composite Hydrogels for Wound Healing. ACS Appl. Mater. Interfaces.

[61] Nesrinne S., Djamel A. (2017). Synthesis, characterization and rheological behavior of pH sensitive poly (acrylamide-co-acrylic acid) hydrogels. Arab. J. Chem..

[62] Ohmine I., Tanaka T. (1982). Salt effects on the phase transition of ionic gels. J. Chem. Phys..

[63] Peterson D.S. (2014). pH-Sensitive Hydrogel. Encyclopedia of Microfluidics and Nanofluidics.

[64] Zhou X., Wang J., Nie J., Du B. (2016). Poly (N-isopropylacrylamide)-based ionic hydrogels: Synthesis, swelling properties, interfacial adsorption and release of dyes. Polym. J..

[65] Lim Y., Kim S.M., Lee Y., Lee W., Yang T., Lee M., Suh H., Park J. (2001). Cationic hyperbranched poly (amino ester): A novel class of DNA condensing molecule with cationic surface, biodegradable three-dimensional structure, and tertiary amine groups in the interior. J. Am. Chem. Soc..

[66] Pagonis K., Bokias G. (2007). Temperature- and solvent-sensitive hydrogels based on *N*-isopropylacrylamide and *N*,*N*-dimethylacrylamide. Polym. Bull..

[67] Zhang X.Z., Yang Y.Y., Chung T.S. (2002). Effect of Mixed Solvents on Characteristics of Poly (N-isopropylacrylamide) Gels. Langmuir.

[68] Chang D.P., Dolbow J.E., Zauscher S. (2007). Switchable Friction of Stimulus-Responsive Hydrogels. Langmuir.

[69] Lu Z.R., Kopečková P., Kopeček J. (2003). Antigen Responsive Hydrogels Based on Polymerizable Antibody Fab Fragment. Macromol. Biosci..

[70] Thornton P.D., Mart R.J., Ulijn R.V. (2007). Enzyme-Responsive Polymer Hydrogel Particles for Controlled Release. Adv. Mater..

[71] Culver H.R., Clegg J.R., Peppas N.A. (2017). Analyte-Responsive Hydrogels: Intelligent Materials for Biosensing and Drug Delivery. Acc. Chem. Res..

[72] Abul-Haija Y.M., Ulijn R.V. (2014). Enzyme-Responsive Hydrogels for Biomedical Applications.

[73] Koetting M.C., Peters J.T., Steichen S.D., Peppas N.A. (2015). Stimulus-responsive hydrogels: Theory, modern advances, and applications. Mater. Sci. Eng. R Rep..

[74] Nazar H., Fatouros D.G., van der Merwe S.M., Bouropoulos N., Avgouropoulos G., Tsibouklis J., Roldo M. (2011). Thermosensitive hydrogels for nasal drug delivery: The formulation and characterisation of systems based on N-trimethyl chitosan chloride. Eur. J. Pharm. Biopharm..

[75] Vihola H., Laukkanen A., Tenhu H., Hirvonen J. (2008). Drug release characteristics of physically cross-linked thermosensitive poly(N-vinylcaprolactam) hydrogel particles. J. Pharm. Sci..

[76] Tan R., She Z., Wang M., Fang Z., Liu Y., Feng Q. (2012). Thermo-sensitive alginate-based injectable hydrogel for tissue engineering. Carbohydr. Polym..

[77] Sala R.L., Kwon M.Y., Kim M., Gullbrand S.E., Henning E.A., Mauck R.L., Camargo E.R., Burdick J.A. (2017). Thermosensitive Poly (N-vinylcaprolactam) Injectable Hydrogels for Cartilage Tissue Engineering. Tissue Eng. Part A.

[78] Fu T.S., Wei Y.H., Cheng P.Y., Chu I.M., Chen W.C. (2018). A Novel Biodegradable and Thermosensitive Poly (Ester-Amide) Hydrogel for Cartilage Tissue Engineering. Biomed. Res. Int..

[79] Klouda L., Mikos A.G. (2008). Thermoresponsive hydrogels in biomedical applications. Eur. J. Pharm. Biopharm..

[80] Gong C., Qi T., Wei X., Qu Y., Wu Q., Luo F., Qian Z. (2013). Thermosensitive polymeric hydrogels as drug delivery systems. Curr. Med. Chem..

[81] Zhang Y., Yu J., Ren K., Zuo J., Ding J., Chen X. (2019). Thermosensitive Hydrogels as Scaffolds for Cartilage Tissue Engineering. Biomacromolecules.

[82] Tahrir F.G., Ganji F., Ahooyi T.M. (2015). Injectable thermosensitive chitosan/glycerophosphate-based hydrogels for tissue engineering and drug delivery applications: A review. Recent Pat. Drug Deliv..

[83] Ge J., Neofytou E., Cahill T.J., Beygui R.E., Zare R.N. (2012). Drug Release from Electric-Field-Responsive Nanoparticles. ACS Nano.

[84] Liu Y., Servant A., Guy O.J., Al-Jamal K.T., Williams P.R., Hawkins K.M., Kostarelos K. (2011). An Electric-Field Responsive Microsystem for Controllable Miniaturised Drug Delivery Applications. Procedia Eng..

[85] Zhao W., Odelius K., Edlund U., Zhao C., Albertsson A.C. (2015). In Situ Synthesis of Magnetic Field-Responsive Hemicellulose Hydrogels for Drug Delivery. Biomacromolecules.

[86] Araújo-Custódio S., Gomez-Florit M., Tomás A.R., Mendes B.B., Babo P.S., Mithieux S.M., Weiss A., Domingues R.M.A., Reis R.L., Gomes M.E. (2019). Injectable and Magnetic Responsive Hydrogels with Bioinspired Ordered Structures. ACS Biomater. Sci. Eng..

[87] Filipcsei G., Csetneki I., Szilágyi A., Zrínyi M. (2007). Magnetic Field-Responsive Smart Polymer Composites. Oligomers-Polymer Composites-Molecular Imprinting.

[88] Ilg P. (2013). Stimuli-responsive hydrogels cross-linked by magnetic nanoparticles. Soft Matter.

[89] El-Leithy E.S., Shaker D.S., Ghorab M.K., Abdel-Rashid R.S. (2010). Evaluation of Mucoadhesive Hydrogels Loaded with Diclofenac Sodium–Chitosan Microspheres for Rectal Administration. AAPS PharmSciTech.

[90] Liu Q., Liu L. (2019). Novel Light-Responsive Hydrogels with Antimicrobial and Antifouling Capabilities. Langmuir.

[91] Li L., Scheiger J.M., Levkin P.A. (2019). Design and Applications of Photoresponsive Hydrogels. Adv. Mater..

[92] ter Schiphorst J., Coleman S., Stumpel J.E., Ben Azouz A., Diamond D., Schenning A.P.H.J. (2015). Molecular Design of Light-Responsive Hydrogels, For in Situ Generation of Fast and Reversible Valves for Microfluidic Applications. Chem. Mater..

[93] Baït N., Grassl B., Derail C., Benaboura A. (2011). Hydrogel nanocomposites as pressure-sensitive adhesives for skin-contact applications. Soft Matter.

[94] Ilic-Stojanovic S., Nikolic L., Nikolic V., Petrovic S., Stankovic M., Mladenovic-Ranisavljevic I. (2011). Stimuli-sensitive hydrogels for pharmaceutical and medical applications. Facta Univ. Ser. Phys. Chem. Technol..

[95] Peppas N.A., Van Blarcom D.S. (2016). Hydrogel-based biosensors and sensing devices for drug delivery. J. Control. Release.

[96] Qiu Y., Park K. (2001). Environment-sensitive hydrogels for drug delivery. Adv. Drug Deliv. Rev..

[97] Bhattacharya M., Malinen M.M., Lauren P., Lou Y.R., Kuisma S.W., Kanninen L., Lille M., Corlu A., GuGuen-Guillouzo C., Ikkala O. (2012). Nanofibrillar cellulose hydrogel promotes three-dimensional liver cell culture. J. Control. Release.

[98] Fu Y., Xu K., Zheng X., Giacomin A.J., Mix A.W., Kao W.J. (2012). 3D cell entrapment in crosslinked thiolated gelatin-poly(ethylene glycol) diacrylate hydrogels. Biomaterials.

[99] Lin R.Z., Chen Y.C., Moreno-Luna R., Khademhosseini A., Melero-Martin J.M. (2013). Transdermal regulation of vascular network bioengineering using a photopolymerizable methacrylated gelatin hydrogel. Biomaterials.

[100] Pok S., Myers J.D., Madihally S.V., Jacot J.G. (2013). A multilayered scaffold of a chitosan and gelatin hydrogel supported by a PCL core for cardiac tissue engineering. Acta Biomater..

[101] Singh M.R., Patel S., Singh D. (2016). Natural polymer-based hydrogels as scaffolds for tissue engineering. Nanobiomaterials in Soft Tissue Engineering.

[102] Chai Q., Jiao Y., Yu X. (2017). Hydrogels for Biomedical Applications: Their Characteristics and the Mechanisms behind Them. Gels.

[103] Vieira S., da Silva Morais A., Silva-Correia J., Oliveira J.M., Reis R.L. (2017). Natural-Based Hydrogels: From Processing to Applications. Encyclopedia of Polymer Science and Technology.

[104] Zhu J., Marchant R.E. (2011). Design properties of hydrogel tissue-engineering scaffolds. Expert Rev. Med. Devices.

[105] Pereira D.R., Canadas R.F., Silva-Correia J., Marques A.P., Reis R.L., Oliveira J.M. (2013). Gellan Gum-Based Hydrogel Bilayered Scaffolds for Osteochondral Tissue Engineering. Key Eng. Mater..

[106] Milašinović N., Kalagasidis Krušić M., Knežević-Jugović Z., Filipović J. (2010). Hydrogels of N-isopropylacrylamide copolymers with controlled release of a model protein. Int. J. Pharm..

[107] Anghelache A., Teodorescu M., Stǎnescu P.O., Drǎghici C., Vuluga D.M. (2014). Novel crosslinked thermoresponsive hydrogels with controlled poly (ethylene glycol)—Poly (propylene glycol) multiblock copolymer structure. Colloid Polym. Sci..

[108] Geckil H., Xu F., Zhang X., Moon S., Demirci U. (2010). Engineering hydrogels as extracellular matrix mimics. Nanomedicine.

[109] Stagnaro P., Schizzi I., Utzeri R., Marsano E., Castellano M. (2018). Alginate-polymethacrylate hybrid hydrogels for potential osteochondral tissue regeneration. Carbohydr. Polym..

[110] Anjum F., Lienemann P.S., Metzger S., Biernaskie J., Kallos M.S., Ehrbar M. (2016). Enzyme responsive GAG-based natural-synthetic hybrid hydrogel for tunable growth factor delivery and stem cell differentiation. Biomaterials.

[111] Huang J., Ren J., Chen G., Li Z., Liu Y., Wang G., Wu X. (2018). Tunable sequential drug delivery system based on chitosan/hyaluronic acid hydrogels and PLGA microspheres for management of non-healing infected wounds. Mater. Sci. Eng. C.

[112] Jia X., Kiick K.L. (2009). Hybrid Multicomponent Hydrogels for Tissue Engineering. Macromol. Biosci..

[113] Aigner T., Stöve J. (2003). Collagens--major component of the physiological cartilage matrix, major target of cartilage degeneration, major tool in cartilage repair. Adv. Drug Deliv. Rev..

[114] Kopecek J. (2003). Smart and genetically engineered biomaterials and drug delivery systems. Eur. J. Pharm. Sci..

[115] Huang Y., Onyeri S., Siewe M., Moshfeghian A., Madihally S.V. (2005). In vitro characterization of chitosan–gelatin scaffolds for tissue engineering. Biomaterials.

[116] Minhas M.U., Ahmad M., Anwar J., Khan S. (2018). Synthesis and Characterization of Biodegradable Hydrogels for Oral Delivery of 5-Fluorouracil Targeted to Colon: Screening with Preliminary In Vivo Studies. Adv. Polym. Technol..

[117] Tan H., Marra K.G. (2010). Injectable, Biodegradable Hydrogels for Tissue Engineering Applications. Materials.

[118] Nguyen M.K., Lee D.S. (2010). Injectable Biodegradable Hydrogels. Macromol. Biosci..

[119] Kitagawa H., Takeda K., Kitagawa R., Izutani N., Miki S., Hirose N., Hayashi M., Imazato S. (2014). Development of sustained antimicrobial-release systems using poly(2-hydroxyethyl methacrylate)/trimethylolpropane trimethacrylate hydrogels. Acta Biomater..

[120] de Cássia Novaes W., Berg A. (2003). Experiences with a New Nonbiodegradable Hydrogel (Aquamid): A Pilot Study. Aesthetic Plast Surg.

[121] Pérez-Luna V., González-Reynoso O. (2018). Encapsulation of Biological Agents in Hydrogels for Therapeutic Applications. Gels.

[122] Lin C.C., Metters A.T. (2006). Hydrogels in controlled release formulations: Network design and mathematical modeling. Adv. Drug Deliv. Rev..

[123] Wong R.S.H., Dodou K. (2017). Effect of Drug Loading Method and Drug Physicochemical Properties on the Material and Drug Release Properties of Poly (Ethylene Oxide) Hydrogels for Transdermal Delivery. Polymers.

[124] Prince D.A., Villamagna I.J., Hopkins C.C., de Bruyn J.R., Gillies E.R. (2019). Effect of drug loading on the properties of temperature-responsive polyester–poly(ethylene glycol)–polyester hydrogels. Polym. Int..

[125] Boca S., Berce C., Jurj A., Petrushev B., Pop L., Gafencu G.A., Selicean S., Moisoiu V., Temian D., Micu W.T. (2017). Ruxolitinib-conjugated gold nanoparticles for topical administration: An alternative for treating alopecia?. Med. Hypotheses.

[126] Allen T.M., Cullis P.R. (2013). Liposomal drug delivery systems: From concept to clinical applications. Adv. Drug Deliv. Rev..

[127] Kataoka K., Harada A., Nagasaki Y. (2012). Block copolymer micelles for drug delivery: Design, characterization and biological significance. Adv. Drug Deliv. Rev..

[128] Pan L., He Q., Liu J., Chen Y., Ma M., Zhang L., Shi J. (2012). Nuclear-Targeted Drug Delivery of TAT Peptide-Conjugated Monodisperse Mesoporous Silica Nanoparticles. J. Am. Chem. Soc..

[129] Jurj A., Braicu C., Pop L.A., Tomuleasa C., Gherman C., Berindan-Neagoe I. (2017). The new era of nanotechnology, an alternative to change cancer treatment. Drug Des. Devel. Ther..

[130] Riggio C., Pagni E., Raffa V., Cuschieri A. (2011). Nano-Oncology: Clinical Application for Cancer Therapy and Future Perspectives. J. Nanomater..

[131] Schexnailder P., Schmidt G. (2009). Nanocomposite polymer hydrogels. Colloid Polym. Sci..

[132] Appel E.A., Tibbitt M.W., Webber M.J., Mattix B.A., Veiseh O., Langer R. (2015). Self-assembled hydrogels utilizing polymer–nanoparticle interactions. Nat. Commun..

[133] Bahari Javan N., Montazeri H., Rezaie Shirmard L., Jafary Omid N., Barbari G.R., Amini M., Ghahremani M.H., Rafiee-Tehrani M., Abedin Dorkoosh F. (2017). Preparation, characterization and in vivo evaluation of a combination delivery system based on hyaluronic acid/jeffamine hydrogel loaded with PHBV/PLGA blend nanoparticles for prolonged delivery of Teriparatide. Eur. J. Pharm. Sci..

[134] Pozzo D.C., Walker L.M. (2007). Shear Orientation of Nanoparticle Arrays Templated in a Thermoreversible Block Copolymer Micellar Crystal. Macromolecules.

[135] Gao W., Zhang Y., Zhang Q., Zhang L. (2016). Nanoparticle-Hydrogel: A Hybrid Biomaterial System for Localized Drug Delivery. Ann. Biomed. Eng..

[136] Baumann M.D., Kang C.E., Tator C.H., Shoichet M.S. (2010). Intrathecal delivery of a polymeric nanocomposite hydrogel after spinal cord injury. Biomaterials.

[137] Gao W., Vecchio D., Li J., Zhu J., Zhang Q., Fu V., Li J., Thamphiwatana S., Lu D., Zhang L. (2014). Hydrogel Containing Nanoparticle-Stabilized Liposomes for Topical Antimicrobial Delivery. ACS Nano.

[138] Jung H.J., Abou-Jaoude M., Carbia B.E., Plummer C., Chauhan A. (2013). Glaucoma therapy by extended release of timolol from nanoparticle loaded silicone-hydrogel contact lenses. J. Control. Release.

[139] Wang F., Gao W., Thamphiwatana S., Luk B.T., Angsantikul P., Zhang Q., Hu C.M.J., Fang R.H., Copp J.A., Pornpattananangkul D. (2015). Hydrogel Retaining Toxin-Absorbing Nanosponges for Local Treatment of Methicillin-Resistant Staphylococcus aureus Infection. Adv. Mater..

[140] Rahman M.S., Islam M.M., Islam M.S., Zaman A., Ahmed T., Biswas S., Sharmeen S., Rashid T.U., Rahman M.M., Mondal M. (2019). Morphological Characterization of Hydrogels. Cellulose-Based Superabsorbent Hydrogels.

[141] Linnes M.P., Ratner B.D., Giachelli C.M. (2007). A fibrinogen-based precision microporous scaffold for tissue engineering. Biomaterials.

[142] Shen J., Yan B., Li T., Long Y., Li N., Ye M. (2012). Study on graphene-oxide-based polyacrylamide composite hydrogels. Compos. Part A Appl. Sci. Manuf..

[143] Aston R., Sewell K., Klein T., Lawrie G., Grøndahl L. (2016). Evaluation of the impact of freezing preparation techniques on the characterisation of alginate hydrogels by cryo-SEM. Eur. Polym. J..

[144] McMahon R., Hahn M., Pendleton M., Ellis E. (2010). A Simple Preparation Method for Mesh Fibrin Hydrogel Composites for Conventional SEM. Microsc. Microanal..

[145] Barker K., Rastogi S.K., Dominguez J., Cantu T., Brittain W., Irvin J., Betancourt T. (2016). Biodegradable DNA-enabled poly(ethylene glycol) hydrogels prepared by copper-free click chemistry. J. Biomater. Sci. Polym. Ed..

[146] Soares P.A.G., CDe Seixas J.R.P., Albuquerque P.B.S., Santos G.R.C., Mourão P.A.S., Barros W., Correia M.T.S., Carneiro-Da-Cunha M.G. (2015). Development and characterization of a new hydrogel based on galactomannan and κ-carrageenan. Carbohydr. Polym..

[147] Treesuppharat W., Rojanapanthu P., Siangsanoh C., Manuspiya H., Ummartyotin S. (2017). Synthesis and characterization of bacterial cellulose and gelatin-based hydrogel composites for drug-delivery systems. Biotechnol. Rep..

[148] Fergg F., Keil F.J., Quader H. (2001). Investigations of the microscopic structure of poly(vinyl alcohol) hydrogels by confocal laser scanning microscopy. Colloid Polym. Sci..

[149] Watkins A.W., Southard S.L., Anseth K.S. (2007). Characterizing multilaminated hydrogels with spatially varying network structure and solute loading using confocal laser scanning microscopy. Acta Biomater..

[150] Watkins A.W., Anseth K.S. (2005). Investigation of molecular transport and distributions in poly(ethylene glycol) hydrogels with confocal laser scanning microscopy. Macromolecules.

[151] Belmar L., Toledo L., Sánchez S.A., Urbano B.F. (2018). Fluorescent nanotubes in PHEMA hydrogels: Visualizing aggregation and distribution by confocal fluorescence microscopy. Mater. Today Commun..

[152] Engler A.J., Sen S., Sweeney H.L., Discher D.E. (2006). Matrix elasticity directs stem cell lineage specification. Cell.

[153] Wong J.Y., Velasco A., Rajagopalan P., Pham Q. (2003). Directed movement of vascular smooth muscle cells on gradient-compliant hydrogels. Langmuir.

[154] Kloxin A.M., Kloxin C.J., Bowman C.N., Anseth K.S. (2010). Mechanical properties of cellularly responsive hydrogels and their experimental determination. Adv. Mater..

[155] Zustiak S.P., Boukari H., Leach J.B. (2010). Solute diffusion and interactions in cross-linked poly(ethylene glycol) hydrogels studied by Fluorescence Correlation Spectroscopy. Soft Matter.

[156] Abuelfilat A.Y., Kim Y., Miller P., Hoo S.P., Li J., Chan P., Fu J. (2015). Bridging structure and mechanics of three-dimensional porous hydrogel with X-ray ultramicroscopy and atomic force microscopy. RSC Adv..

[157] Cocarta A.I., Hobzova R., Sirc J., Cerna T., Hrabeta J., Svojgr K., Pochop P., Kodetova M., Jedelska J., Bakowsky U. (2019). Hydrogel implants for transscleral drug delivery for retinoblastoma treatment. Mater. Sci. Eng. C.

[158] Longchamp A., Kaur K., Macabrey D., Dubuis C., Corpataux J.M., Déglise S., Matson J.B., Allagnat F. (2019). Hydrogen Sulfide-releasing peptide hydrogel limits the development of intimal hyperplasia in human vein segments. Acta Biomater..

[159] Ishihara M., Obara K., Ishizuka T., Fujita M., Sato M., Masuoka K., Saito Y., Yura H., Matsui T., Hattori H. (2003). Controlled release of fibroblast growth factors and heparin from photocrosslinked chitosan hydrogels and subsequent effect onin vivo vascularization. J. Biomed. Mater. Res..

[160] Um S.H., Lee J.B., Park N., Kwon S.Y., Umbach C.C., Luo D. (2006). Enzyme-catalysed assembly of DNA hydrogel. Nat. Mater..

[161] Lutolf M.P., Lauer-Fields J.L., Schmoekel H.G., Metters A.T., Weber F.E., Fields G.B., Hubbell J.A. (2003). Synthetic matrix metalloproteinase-sensitive hydrogels for the conduction of tissue regeneration: Engineering cell-invasion characteristics. Proc. Natl. Acad. Sci. USA.

[162] Podual K., Doyle F.J., Peppas N.A. (2000). Glucose-sensitivity of glucose oxidase-containing cationic copolymer hydrogels having poly (ethylene glycol) grafts. J. Control. Release.

[163] Maitz M.F., Freudenberg U., Tsurkan M.V., Fischer M., Beyrich T., Werner C. (2013). Bio-responsive polymer hydrogels homeostatically regulate blood coagulation. Nat. Commun..

[164] Yan B., Boyer J.C., Habault D., Branda N.R., Zhao Y. (2012). Near Infrared Light Triggered Release of Biomacromolecules from Hydrogels Loaded with Upconversion Nanoparticles. J. Am. Chem. Soc..

[165] Zhang Y., Wang R., Hua Y., Baumgartner R., Cheng J. (2014). Trigger-Responsive Poly(β-amino ester) Hydrogels. ACS Macro Lett..

[166] Caccavo D., Cascone S., Lamberti G., Barba A.A., Larsson A. (2016). Swellable Hydrogel-based Systems for Controlled Drug Delivery. Smart Drug Delivery System.

[167] Chen Y., Liu W.Y., Zeng G.S. (2016). Stimulus-responsive hydrogels reinforced by cellulose nanowhisker for controlled drug release. RSC Adv..

[168] Liu T.Y., Hu S.H., Liu T.Y., Liu D.M., Chen S.Y. (2006). Magnetic-Sensitive Behavior of Intelligent Ferrogels for Controlled Release of Drug. Langmuir.

[169] Huebsch N., Kearney C.J., Zhao X., Kim J., Cezar C.A., Suo Z., Mooney D.J. (2014). Ultrasound-triggered disruption and self-healing of reversibly cross-linked hydrogels for drug delivery and enhanced chemotherapy. Proc. Natl. Acad. Sci. USA.

[170] Caccavo D., Cascone S., Lamberti G., Barba A.A. (2015). Modeling the Drug Release from Hydrogel-Based Matrices. Mol. Pharm..

[171] Han T.S., Hur K., Choi B., Lee J.Y., Byeon S.J., Min J., Yu J., Cho J.K., Hong J., Lee H.J. (2017). Improvement of anti-cancer drug efficacy via thermosensitive hydrogel in peritoneal carcinomatosis in gastric cancer. Oncotarget.

[172] Naahidi S., Jafari M., Logan M., Wang Y., Yuan Y., Bae H., Dixon B., Chen P. (2017). Biocompatibility of hydrogel-based scaffolds for tissue engineering applications. Biotechnol. Adv..

[173] Palmer B., DeLouise L., Palmer B.C., DeLouise L.A. (2016). Nanoparticle-Enabled Transdermal Drug Delivery Systems for Enhanced Dose Control and Tissue Targeting. Molecules.

[174] Gomes M., Azevedo H., Malafaya P., Silva S., Oliveira J., Silva G., Sousa R., Mano J., Reis R. (2008). Natural Polymers in tissue engineering applications. Tissue Eng..

[175] Ma X., Sun X., Hargrove D., Chen J., Song D., Dong Q., Lu X., Fan T.H., Fu Y., Lei Y. (2016). A Biocompatible and Biodegradable Protein Hydrogel with Green and Red Autofluorescence: Preparation, Characterization and In Vivo Biodegradation Tracking and Modeling. Sci. Rep..

[176] Lin H.A., Varma D.M., Hom W.W., Cruz M.A., Nasser P.R., Phelps R.G., Iatridis J.C., Nicoll S.B. (2019). Injectable cellulose-based hydrogels as nucleus pulposus replacements: Assessment of in vitro structural stability, ex vivo herniation risk, and in vivo biocompatibility. J. Mech. Behav. Biomed. Mater..

[177] Hyun H., Kim Y.H., Song I.B., Lee J.W., Kim M.S., Khang G., Park K., Lee H.B. (2007). In Vitro and in Vivo Release of Albumin Using a Biodegradable MPEG-PCL Diblock Copolymer as an in Situ Gel-Forming Carrier. Biomacromolecules.

[178] Wang Y., Chen L., Tan L., Zhao Q., Luo F., Wei Y., Qian Z. (2014). PEG–PCL based micelle hydrogels as oral docetaxel delivery systems for breast cancer therapy. Biomaterials.

[179] Ramadan A.A., Elbakry A.M., Esmaeil A.H., Khaleel S.A. (2018). Pharmaceutical and pharmacokinetic evaluation of novel rectal mucoadhesive hydrogels containing tolmetin sodium. J. Pharm. Investig..

[180] Bhaskar K., Mohan C.K., Lingam M., Mohan S.J., Venkateswarlu V., Rao Y.M., Bhaskar K., Anbu J., Ravichandran V. (2009). Development of SLN and NLC Enriched Hydrogels for Transdermal Delivery of Nitrendipine: In Vitro and In Vivo Characteristics. Drug Dev. Ind. Pharm..

[181] Wu Q., Wang N., He T., Shang J., Li L., Song L., Yang X., Li X., Luo N., Zhang W. (2015). Thermosensitive hydrogel containing dexamethasone micelles for preventing postsurgical adhesion in a repeated-injury model. Sci. Rep..

[182] Ohta S., Hiramoto S., Amano Y., Emoto S., Yamaguchi H., Ishigami H., Kitayama J., Ito T. (2017). Intraperitoneal Delivery of Cisplatin via a Hyaluronan-Based Nanogel/ in Situ Cross-Linkable Hydrogel Hybrid System for Peritoneal Dissemination of Gastric Cancer. Mol. Pharm..

[183] Hosny K.M. (2010). Ciprofloxacin as Ocular Liposomal Hydrogel. AAPS PharmSciTech.

[184] Schellini S.A., Zimmermann G.P.M., Hoyama E., Pellizon C.H., Padovani C.R., Selva D. (2008). Polyethylene Gel in the Subcutaneous Tissue of Rats: Histopathologic and Systemic Evaluation. Orbit.

[185] Sharma G., Italia J.L., Sonaje K., Tikoo K., Ravi Kumar M.N.V. (2007). Biodegradable in situ gelling system for subcutaneous administration of ellagic acid and ellagic acid loaded nanoparticles: Evaluation of their antioxidant potential against cyclosporine induced nephrotoxicity in rats. J. Control. Release.

[186] Takahashi M., Heo Y.J., Shibata H., Satou H., Kawanishi T., Okitsu T., Takeuchi S. Nano-patterned hydrogel reduced inflammatory effects in subcutaneous tissue. Proceedings of the 2012 IEEE 25th International Conference on Micro Electro Mechanical Systems (MEMS).

[187] Hou H.Y., Fu S.H., Liu C.H., Chen J.P., Ray-Sea Hsu B. (2014). The graft survival protection of subcutaneous allogeneic islets with hydrogel grafting and encapsulated by CTLA4Ig and IL1ra. Polym. J..

[188] Bae J.H., Shrestha K.R., Park Y.H., Kim I.G., Piao S., Jung A.R., Jeon S.H., Park K.D., Lee J.Y. (2014). Comparison between subcutaneous injection of basic fibroblast growth factor-hydrogel and intracavernous injection of adipose-derived stem cells in a rat model of cavernous nerve injury. Urology.

[189] Halberstadt C., Austin C., Rowley J., Culberson C., Loebsack A., Wyatt S., Coleman S., Blacksten L., Burg K., Mooney D. (2002). A Hydrogel Material for Plastic and Reconstructive Applications Injected into the Subcutaneous Space of a Sheep. Tissue Eng..

[190] Markov P.A., Khramova D.S., Shumikhin K.V., Nikitina I.R., Beloserov V.S., Martinson E.A., Litvinets S.G., Popov S.V. (2019). Mechanical properties of the pectin hydrogels and inflammation response to their subcutaneous implantation. J. Biomed. Mater. Res. Part A.

[191] Khafagy E.S., Morishita M., Onuki Y., Takayama K. (2007). Current challenges in non-invasive insulin delivery systems: A comparative review. Adv. Drug Deli Rev..

[192] Tulain U.R., Ahmad M., Rashid A., Malik M.Z., Iqbal F.M. (2018). Fabrication of pH-Responsive Hydrogel and Its In Vitro and In Vivo Evaluation. Adv. Polym. Technol..

[193] Tan L., Xu X., Song J., Luo F., Qian Z. (2013). Synthesis, characterization, and acute oral toxicity evaluation of pH-sensitive hydrogel based on MPEG, poly (ε-caprolactone), and itaconic acid. Biomed. Res. Int..

[194] Sharpe L.A., Daily A.M., Horava S.D., Peppas N.A. (2014). Therapeutic applications of hydrogels in oral drug delivery. Expert Opin. Drug Deliv..

[195] Kim K., Kim K., Ryu J.H., Lee H. (2015). Chitosan-catechol: A polymer with long-lasting mucoadhesive properties. Biomaterials.

[196] Xu J., Tam M., Samaei S., Lerouge S., Barralet J., Stevenson M.M., Cerruti M. (2017). Mucoadhesive chitosan hydrogels as rectal drug delivery vessels to treat ulcerative colitis. Acta Biomater..

[197] Chakrabarti S., Islam J., Hazarika H., Mazumder B., Raju P.S., Chattopadhyay P. (2018). Safety profile of silver sulfadiazine-bFGF-loaded hydrogel for partial thickness burn wounds. Cutan. Ocul. Toxicol..

[198] Poh Yuen Wen A., Halim A.S., Mat Saad A.Z., Mohd Nor F., Wan Sulaiman W.A. (2018). A prospective study evaluating wound healing with sea cucumber gel compared with hydrogel in treatment of skin graft donor sites. Complement. Med..

[199] Fernandes R., Smyth N.R., Muskens O.L., Nitti S., Heuer-Jungemann A., Ardern-Jones M.R., Kanaras A.G. (2015). Interactions of Skin with Gold Nanoparticles of Different Surface Charge, Shape, and Functionality. Small.

[200] Basso J., Miranda A., Nunes S., Cova T., Sousa J., Vitorino C., Pais A., Basso J., Miranda A., Nunes S. (2018). Hydrogel-Based Drug Delivery Nanosystems for the Treatment of Brain Tumors. Gels.

[201] Zhao M., Danhier F., Bastiancich C., Joudiou N., Ganipineni L.P., Tsakiris N., Gallez B., des Rieux A., Jankovski A., Bianco J. (2018). Post-resection treatment of glioblastoma with an injectable nanomedicine-loaded photopolymerizable hydrogel induces long-term survival. Int. J. Pharm..

[202] Bastiancich C., Bianco J., Vanvarenberg K., Ucakar B., Joudiou N., Gallez B., Bastiat G., Lagarce F., Préat V., Danhier F. (2017). Injectable nanomedicine hydrogel for local chemotherapy of glioblastoma after surgical resection. J. Control. Release.

[203] Chen C.H., Kuo C.Y., Chen S.H., Mao S.H., Chang C.Y., Shalumon K., Chen J.P., Chen C.H., Kuo C.Y., Chen S.H. (2018). Thermosensitive Injectable Hydrogel for Simultaneous Intraperitoneal Delivery of Doxorubicin and Prevention of Peritoneal Adhesion. Int. J. Mol. Sci..

[204] Yamashita K., Tsunoda S., Gunji S., Murakami T., Suzuki T., Tabata Y., Sakai Y. (2019). Intraperitoneal chemotherapy for peritoneal metastases using sustained release formula of cisplatin-incorporated gelatin hydrogel granules. Surg. Today.

[205] Mandal A., Bisht R., Rupenthal I.D., Mitra A.K. (2017). Polymeric micelles for ocular drug delivery: From structural frameworks to recent preclinical studies. J. Control. Release.

[206] Bisht R., Jaiswal J.K., Chen Y.S., Jin J., Rupenthal I.D. (2016). Light-responsive in situ forming injectable implants for effective drug delivery to the posterior segment of the eye. Expert Opin. Drug Deliv..

[207] Jacob J.T. (2013). Biocompatibility in the Development of Silicone-Hydrogel Lenses. Eye Contact Lens Sci. Clin. Pract..

[208] Hall B.J., Jones L.W., Dixon B. (2014). Silicone allergies and the eye: Fact or fiction?. Eye Contact Lens.

[209] Liu M., Zeng X., Ma C., Yi H., Ali Z., Mou X., Li S., Deng Y., He N. (2017). Injectable hydrogels for cartilage and bone tissue engineering. Bone Res..

[210] Budama-Kilinc Y., Cakir-Koc R., Aslan B., Özkan B., Mutlu H., Üstün E. (2018). Hydrogels in Regenerative Medicine. Biomaterials in Regenerative Medicine.

[211] Gutowska A., Jeong B., Jasionowski M. (2001). Injectable gels for tissue engineering. Anat. Rec..

[212] Kofron M.D., Laurencin C.T. (2006). Bone tissue engineering by gene delivery. Adv. Drug Deliv. Rev..

[213] Hong Y., Gong Y., Gao C., Shen J. (2008). Collagen-coated polylactide microcarriers/chitosan hydrogel composite: Injectable scaffold for cartilage regeneration. J. Biomed. Mater. Res. A.

[214] Jin R., Moreira Teixeira L.S., Dijkstra P.J., Karperien M., van Blitterswijk C.A., Zhong Z.Y., Feijen J. (2009). Injectable chitosan-based hydrogels for cartilage tissue engineering. Biomaterials.

[215] Shen Z.S., Cui X., Hou R.X., Li Q., Deng H.X., Fu J. (2015). Tough biodegradable chitosan–gelatin hydrogels via in situ precipitation for potential cartilage tissue engineering. RSC Adv..

[216] Park H., Lee K.Y. (2014). Cartilage regeneration using biodegradable oxidized alginate/hyaluronate hydrogels. J. Biomed. Mater. Res. A.

[217] Zhao X., Liu S., Yildirimer L., Zhao H., Ding R., Wang H., Cui W., Weitz D. (2016). Injectable Stem Cell-Laden Photocrosslinkable Microspheres Fabricated Using Microfluidics for Rapid Generation of Osteogenic Tissue Constructs. Adv. Funct. Mater..

[218] Perrier-Groult E., Pérès E., Pasdeloup M., Gazzolo L., Duc Dodon M., Mallein-Gerin F. (2019). Evaluation of the biocompatibility and stability of allogeneic tissue-engineered cartilage in humanized mice. PLoS ONE.

[219] Patzelt A., Mak W.C., Jung S., Knorr F., Meinke M.C., Richter H., Rühl E., Cheung K.Y., Tran N.B.N.N., Lademann J. (2017). Do nanoparticles have a future in dermal drug delivery?. J. Control. Release.

[220] Du Q., Jiang L., Wang X.Q., Pan W., She F.F., Chen Y.L. (2014). Establishment of and comparison between orthotopic xenograft and subcutaneous xenograft models of gallbladder carcinoma. Asian Pac. J. Cancer Prev..

[221] van Gysen K., Kneebone A., Alfieri F., Guo L., Eade T. (2014). Feasibility of and rectal dosimetry improvement with the use of SpaceOAR^®^ hydrogel for dose-escalated prostate cancer radiotherapy. J. Med. Imaging Radiat. Oncol..

[222] Juneja P., Kneebone A., Booth J.T., Thwaites D.I., Kaur R., Colvill E., Ng J.A., Keall P.J., Eade T. (2015). Prostate motion during radiotherapy of prostate cancer patients with and without application of a hydrogel spacer: A comparative study. Radiat. Oncol..

[223] Liu C., Lu Q., Zhang Z., Xue M., Zhang Y., Zhang Y., Wang H., Li H., Zhou Y., Zhang Z. (2015). A Randomized Controlled Trial on the Efficacy and Safety of a New Crosslinked Hyaluronan Gel in Reducing Adhesions after Gynecologic Laparoscopic Surgeries. J. Minim. Invasive Gynecol..

[224] Lin M.C., French H.M., Graham A.D., Sanders T.L. (2014). Effects of Daily Irrigation on Corneal Epithelial Permeability and Adverse Events With Silicone Hydrogel Contact Lens Continuous Wear. Investig. Ophthalmol. Vis. Sci..

[225] Tagliaferri A., Love T.E., Szczotka-Flynn L.B. (2014). Risk Factors for Contact Lens–Induced Papillary Conjunctivitis Associated With Silicone Hydrogel Contact Lens Wear. Eye Contact Lens Sci. Clin. Pract..

[226] Szczotka-Flynn L., Lass J.H., Sethi A., Debanne S., Benetz B.A., Albright M., Gillespie B., Kuo J., Jacobs M.R., Rimm A. (2010). Risk Factors for Corneal Infiltrative Events during Continuous Wear of Silicone Hydrogel Contact Lenses. Investig. Ophthalmol. Vis. Sci..

[227] Varikooty J., Keir N., Richter D., Jones L.W., Woods C., Fonn D. (2013). Comfort Response of Three Silicone Hydrogel Daily Disposable Contact Lenses. Optom. Vis. Sci..

[228] Fallacara A., Vertuani S., Panozzo G., Pecorelli A., Valacchi G., Manfredini S. (2017). Novel Artificial Tears Containing Cross-Linked Hyaluronic Acid: An In Vitro Re-Epithelialization Study. Molecules.

[229] Altman D., Ghilotti F., Bellocco R., Zetterström J., Kopp Kallner H. (2017). Transurethral Polyacrylamide Hydrogel Injection Therapy in Women Not Eligible for Midurethral Sling Surgery. Female Pelvic Med. Reconstr. Surg..

[230] Green A.L., Arnaud A., Batiller J., Eljamel S., Gauld J., Jones P., Martin D., Mehdorn M., Ohman J., Weyns F. (2015). A multicentre, prospective, randomized, controlled study to evaluate the use of a fibrin sealant as an adjunct to sutured dural repair. Br. J. Neurosurg..

[231] Agren M.S. (1998). An amorphous hydrogel enhances epithelialisation of wounds. Acta Derm. Venereol..

[232] Naggara O., Darsaut T., Trystram D., Tselikas L., Raymond J. (2014). Unruptured intracranial aneurysms: Why we must not perpetuate the impasse for another 25 years. Lancet Neurol..

[233] White P.M., Lewis S.C., Gholkar A., Sellar R.J., Nahser H., Cognard C., Forrester L., Wardlaw J.M. (2011). Hydrogel-coated coils versus bare platinum coils for the endovascular treatment of intracranial aneurysms (HELPS): A randomised controlled trial. Lancet.

[234] Kusano T., Etoh T., Akagi T., Ueda Y., Shiroshita H., Yasuda K., Satoh M., Inomata M., Shiraishi N., Kitano S. (2014). Evaluation of 0.6% sodium alginate as a submucosal injection material in endoscopic submucosal dissection for early gastric cancer. Dig. Endosc..

[235] Allison R.R., Ambrad A.A., Arshoun Y., Carmel R.J., Ciuba D.F., Feldman E., Finkelstein S.E., Gandhavadi R., Heron D.E., Lane S.C. (2014). Multi-institutional, randomized, double-blind, placebo-controlled trial to assess the efficacy of a mucoadhesive hydrogel (MuGard) in mitigating oral mucositis symptoms in patients being treated with chemoradiation therapy for cancers of the head and neck. Cancer.

[236] Mettler L., Hucke J., Bojahr B., Tinneberg H.R., Leyland N., Avelar R. (2008). A safety and efficacy study of a resorbable hydrogel for reduction of post-operative adhesions following myomectomy. Hum. Reprod..

[237] Ingenito E.P., Berger R.L., Henderson A.C., Reilly J.J., Tsai L., Hoffman A. (2003). Bronchoscopic Lung Volume Reduction Using Tissue Engineering Principles. Am. J. Respir. Crit. Care Med..

[238] Reilly J., Washko G., Pinto-Plata V., Velez E., Kenney L., Berger R., Celli B. (2007). Biological Lung Volume Reduction. Chest.

[239] Zaetta J.M., Licht M.O., Fisher J.S., Avelar R.L. (2010). A Lung Biopsy Tract Plug for Reduction of Postbiopsy Pneumothorax and Other Complications: Results of a Prospective, Multicenter, Randomized, Controlled Clinical Study. J. Vasc. Interv. Radiol..

[240] Takehara N., Tsutsumi Y., Tateishi K., Ogata T., Tanaka H., Ueyama T., Takahashi T., Takamatsu T., Fukushima M., Komeda M. (2008). Controlled Delivery of Basic Fibroblast Growth Factor Promotes Human Cardiosphere-Derived Cell Engraftment to Enhance Cardiac Repair for Chronic Myocardial Infarction. J. Am. Coll. Cardiol..

[241] Mann D.L., Lee R.J., Coats A.J.S., Neagoe G., Dragomir D., Pusineri E., Piredda M., Bettari L., Kirwan B.-A., Dowling R. (2016). One-year follow-up results from AUGMENT-HF: A multicentre randomized controlled clinical trial of the efficacy of left ventricular augmentation with Algisyl in the treatment of heart failure. Eur. J. Heart Fail..

[242] Anker S.D., Coats A.J.S., Cristian G., Dragomir D., Pusineri E., Piredda M., Bettari L., Dowling R., Volterrani M., Kirwan B.A. (2015). A prospective comparison of alginate-hydrogel with standard medical therapy to determine impact on functional capacity and clinical outcomes in patients with advanced heart failure (AUGMENT-HF trial). Eur. Heart J..

[243] Osbun J.W., Ellenbogen R.G., Chesnut R.M., Chin L.S., Connolly P.J., Cosgrove G.R., Delashaw J.B., Golfinos J.G., Greenlee J.D.W., Haines S.J. (2012). A Multicenter, Single-Blind, Prospective Randomized Trial to Evaluate the Safety of a Polyethylene Glycol Hydrogel (Duraseal Dural Sealant System) as a Dural Sealant in Cranial Surgery. World Neurosurg..

[244] Palladini M., Boesl I., Koenig S., Buchheister B., Attal N. (2019). Lidocaine medicated plaster, an additional potential treatment option for localized post-surgical neuropathic pain: Efficacy and safety results of a randomized, placebo-controlled trial. Curr. Med. Res. Opin..

